# The Ecological Footprint of COVID-19 mRNA Vaccines: Estimating Greenhouse Gas Emissions in Germany

**DOI:** 10.3390/ijerph18147425

**Published:** 2021-07-12

**Authors:** Peter Kurzweil, Alfred Müller, Steffen Wahler

**Affiliations:** 1 Department MBUT, Technical University of Applied Sciences (OTH), Kaiser-Wilhelm-Ring 23, 92224 Amberg, Germany; 2 Analytic Services, Jahnstr. 34c, 80469 München, Germany; alfred.mueller@analytic-services.de; 3 St. Bernward GmbH, Friedrich-Kirsten-Str. 40, 22391 Hamburg, Germany; steffen.wahler@t-online.de

**Keywords:** ecological impact analysis, CO_2_ footprint, COVID-19 vaccine

## Abstract

Compared to the medical, economic and social implications of COVID-19 vaccinations, little attention has been paid to the ecological balance to date. This study is an attempt to estimate the environmental impact of two mRNA vaccines in terms of CO_2_ equivalents with respect to their different freezing strategies and supply chain organization. Although it is impossible to accurately calculate the actual environmental impact of the new biochemical synthesis technology, it becomes apparent that transport accounts for up to 99% of the total carbon footprint. The emissions for air freight, road transportation and last-mile delivery are nearly as 19 times the emissions generated from ultra-deep freeze technologies, the production of dry ice, glass and medical polymers for packaging. The carbon footprint of a single mRNA vaccine dose injected into a patient is about 0.01 to 0.2 kg CO_2_ equivalents, depending on the cooling technology and the logistic routes to the vaccination sites in Germany.

## 1. Introduction

Compared to the medical and economic challenges raised by the pandemic in high-, middle and low-income countries [1], the environmental impact of vaccination programs appears to be a secondary concern. However, misinformation, misrepresentations and various rumors spread by vaccination critics have prompted us to assess the impact of COVID-19 vaccines on climate. The authors are aware that a comprehensive in-depth ecological analysis of the entire process chain from the raw materials to the end user is hardly feasible. Therefore, this analysis focuses on estimating the carbon footprint [2,3] of packaging, distribution and cold chain storage [4,5,6,7] of messenger RNA (mRNA) vaccines. The temporary recovery of global energy demands, carbon and NO_x_ emissions during the lockdown measures [8,9,10,11] has not been considered. 

The groundbreaking mRNA vaccines require multiple highly purified ingredients and complex process steps with yet unknown ecologically relevant material flows and waste streams. RNA production is based on DNA plasmids as templates on which mRNA is built. This new approach is much faster than normal vector vaccine production using infected animal cell cultures or chicken eggs, which take several weeks to incubate. Vector vaccines require living cells while RNA manufacturing is a biochemical process. The enzyme RNA polymerase synthesizes the ribonucleotides that represent the building blocks G, A, U and C of the desired mRNA strand. Co-formulation of the sensitive mRNA molecules into different lipid nanoparticles (e.g., fatty acid esters of tertiary amines, lecithins, cholesterol) facilitates their uptake into cells. Instead of applying a weakened copy of the coronavirus, mRNA strands instruct the body’s immune system cells to develop antibodies against the spike protein of SARS-CoV-2. 

BioNTech BNT162b2 [12,13] is an aqueous concentrate that must be diluted with physiological saline before intramuscular injection. The other ingredients, potassium chloride, sodium chloride, phosphates and sucrose, adjust to a suitable pH and protect the vaccine during the freezing process. The preparation is free of adjuvants or preservatives. One injection vial containing 0.45 mL of concentrate is diluted with 1.8 mL of saline after thawing to obtain ready-to-use 2.25 mL of liquid for six doses of 0.3 mL each (30 µg of active ingredient). An interrupted cold chain, vigorous shaking of diluted vaccine doses and mechanically improper injection will damage the mRNA and weaken the effect of the vaccine [14]. Two injections three to six weeks apart are required to ensure full immunization. The vaccine is manufactured in the United States, Belgium (Puurs) and Germany (Marburg, Mainz, Idar-Oberstein). The frozen undiluted vaccine is required to be stored at temperatures between −80 and −60 °C for up to 6 months. The ultra-low temperature vaccine is delivered directly from BioNTech to wholesalers (−25 to −15 °C for up to 2 weeks), where it is temporarily stored, thawed and delivered to pharmacies refrigerated at 2–8 °C (up to 1 month). At general practitioner offices, the vaccine must be diluted for use within 2 to 6 h (2–25 °C).

The Moderna mRNA-1273 vaccine [15,16] is intended to be administered as two 0.5 mL doses given by intramuscular injection four to six weeks apart. The vaccine contains the modified mRNA, lipids (such as SM-102, polyethylene glycol, cholesterol, phosphocholine derivatives), pH regulators (tromethamine, sodium acetate an others) and sucrose [17]. The vaccine is manufactured at facilities in Portsmouth (GB), New Hampshire (USA) and Visp in Switzerland. Bottling and packaging of the vials takes place in the USA and Spain. Undiluted frozen vials of Moderna vaccine can be transported and stored at −25 to −15 °C for up to four months. Storage at 2–8 °C is possible for up to thirty days. At room temperature, the vaccine must be used within 6 to 12 h (8–25 °C).

Adenovirus vector vaccines have not been further analyzed in this study as they do not require deep and ultra-deep freeze technologies.

## 2. Methodology 

The complete environmental impact of the COVID-19 pandemic is unlikely to be quantifiable in the near future. Therefore, a comparison of packaging, transport routes and cold chains for different mRNA vaccines is calculated based on generally accepted CO_2_ equivalent values. This might answer the question to what extent mRNA vaccines actually generate greenhouse gases and plastic waste.

Germany, the largest vaccination market in Europe, was chosen to calculate the environmental impact of the transport sector. Distances for air freight and road transportation were assumed to cover all process steps from manufacture to the end user. Details on the last-mile simulation are given in Section 3.4.

## 3. Results and Discussion

### 3.1. Production, Sterilization and Waste

Differences in the biochemical production of the various mRNA vaccines are likely, but could not be quantified in this study due to lack of publicly available data. According to BioNTech, 50,000 steps are required to produce the vaccine from the mRNA to the bulk drug substance [18], making it impossible to reliably estimate the carbon footprint. Negative environmental impacts of mRNA vaccination include the manufacturing, use and disposal of polymers and glass, the use of dry ice and freezers and the CO_2_ emissions from the trucks and aircraft needed to deliver the vaccines to millions of patients. Table 1 compiles the data available from the literature to estimate energy expenditures. Carbon footprints in kg CO_2_ equivalents per kg product are in the order of magnitude of (e.g., [19]): 0.07 (steel), 0.3… 0.4 (glass), 0.26…1 (mixed plastics), 0.6… 1.9 (beer), 2.1 (biodiesel), 0.3… 6.3 (biopolymers), 6 (milk powder), 1.8… 11 (aluminum cans), 6… 11 (coffee), 10… 100 (beef) and 52… 572 (biotechnological silk protein).

#### 3.1.1. Pharmaceutical Residues 

Waste prevention fulfills one of the twelve principles of green chemistry. Sheldon’s environmental factor (E = kg waste/kg product) [20,21] places a high value on waste elimination and the avoidance of hazardous substances in organic synthesis. While oil refining generates the highest annual tonnages of waste (E < 0.1), pharmaceutical processes have the largest mass difference between raw materials and high-quality end products (E up to 100 and more). The main challenge with E-factors is that deep knowledge of all stages of the production and product life-cycle is required. Considering the quantities used, the pharmaceutical sector produces a much lower tonnage of waste than any other industry.

Medical waste [22] is growing due to syringes, ampoules, masks, protective suits and shields used in vaccination campaigns. Typically, polypropylene is used for syringes and N-95 masks, and polyethylene for protective suits, gloves and medical face shields. Cyclic olefin polymer (COP) [23] can replace polypropylene and produces the least amount of ash after combustion. Measured against commercial and municipal waste, the additional garbage of a temporary vaccination campaign appears to be insignificant, especially since there are functioning recycling channels for used paper, glass and plastic [24]. However, only 1% of the world’s polypropylene is recycled, and most of it ends up in landfills [25]. In Wuhan (China), for example, during the time of the COVID-19 outbreak, medical waste abruptly increased by 190 t (to 240 t) per day [26]. Unfortunately, the SARS-CoV-2 virus can exist on cardboard for one day and on plastics and steel for up to three days [27], requiring additional waste sorting. Since BNT162b2 contains six doses per vial, and mRNA-1273 contains ten doses in a larger vial, the difference in the eco-balance in terms of glass and medical waste is small, especially as glass vials can be recycled.

#### 3.1.2. Sterilization 

Large energy savings result from energy-efficient equipment used to produce injection-grade water in sterile filling plants. To remove ions, chlorine, particles and endotoxins, the water is filtered, deionized and distilled in clean rooms.

#### 3.1.3. Disinfectants 

The ecological impact of disinfectants was not considered [28].

### 3.2. Freezing and Storage

**Figure 1:**
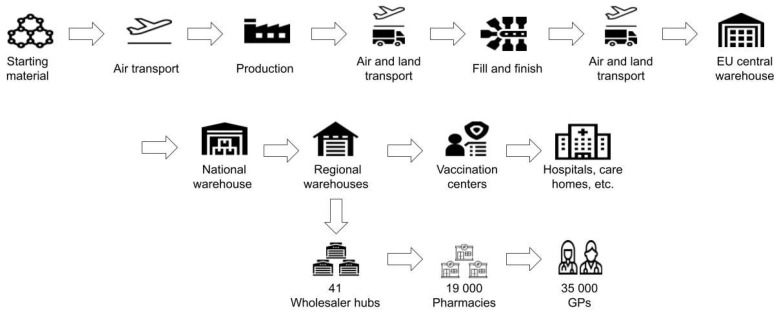
Supply chain and last mile delivery process for mRNA vaccines in Germany.

The COVID-19 vaccine cold chain includes (1) shipping from production facilities to medical trials and drug manufacturers by truck, (2) high-volume refrigerated shipments to global distribution centers by aircraft, (3) distribution to thousands of regional healthcare facilities by truck, and (4) distribution to local healthcare facilities by courier services. Figure 1 illustrates the delivery process of the last mile in Germany (for details, see Section 3.4) The cold chain requires both freezer capacity at the storage and distribution centers and special packaging to maintain the extremely low temperatures between the storage centers. 

#### 3.2.1. Dry Ice 

Vaccines that must be kept at specific temperatures, either between −20 °C and −80 °C or between 2 °C and 8 °C, from the time of filling in the factory to the end user require temperature-controlled packaging, some of which require dry ice as a refrigerant. Dry ice or ‘carbonic acid snow’ is the solid, frozen form of carbon dioxide (−78.5 °C) for shipping biologics without electrical refrigeration units. It sublimates directly from solid to vapor and leaves no residue that could damage packaging or cargo. Compressed CO_2_ in gas cylinders is liquid above 58 bar (20 °C, 0.766 g/cm^3^) and condenses as dry ice when it flows out. The energy required for production of liquid CO_2_ in a refrigeration plant amounts to 0.2 … 0.55 kWh/kg; dry ice from CO_2_ requires 0.17 … 0.32 kWh/kg [38].

Today, carbon dioxide as an industrial gas comes mainly from processed exhaust gases and natural fermentation processes in breweries. Dry ice is derived from CO_2_ generated during ethanol production and the refining of petroleum into gasoline and the combustion of natural gas to produce ammonia, e.g., for fertilizers. CO_2_ waste is generated by burning coke or natural gas (C + O_2_ → CO_2_), carbon monoxide conversion (CO + H_2_O → CO_2_), calcination of lime (CaCO_3_ → CaO + CO_2_) and gas purification with ethanolamine (CO + RNH_2_ + H_2_O → RNH_3_HCO_3_). 

The capture and use of CO_2_ are more or less climate neutral or at least reduce the amount of waste CO_2_ released into the atmosphere. Dry ice accounts for about 20% of the CO_2_ demand in the USA, with an increasing trend from home delivery of frozen foods [39].

As a rule of thumb, 2.3 to 4.5 kg of dry ice sublime every 24 h, depending on the density of the expanded polystyrene foam container [40]. Commercial dry ice boxes have low loss rates of about 1.4 to 5 g/h per liter of volume. Payloads of up to 80 L can be safely stored below −20 °C for 100 h by passive temperature control with vacuum insulation panels.

The production and use of dry ice have a minor impact on the carbon footprint. Dry ice storage does not necessarily have to be a significant energetic disadvantage in every case. Ultra-cooling at −70 °C adds about 0.1 kg CO_2_ equivalents per dose to the CO_2_ balance (see Table 2). Inefficient freezer units and refrigerators shift the CO_2_ balance unfavorably. Refrigerators with poor efficiency cause more emissions than efficient central refrigeration units.

#### 3.2.2. Refrigerators 

Fluorocarbons in refrigerators, which deplete the ozone layer and have a high global warming potential, have largely been replaced by less harmful hydrocarbons in recent decades. Since the refrigeration units are hermetically sealed, the risk of additional HFC pollution from the use of COVID-19 vaccines appears to be negligible. However, cold storage and long-distance transport do not align with the EU’s Green Deal goal of becoming climate neutral by 2050. 

For ultra-cold storage of vaccines (−20 °C to −80 °C), portable and stationary cryostats have been developed. Ultra-cooling units of older designs are less effective (see Table 2).

### 3.3. Transportation and Logistics

The International Air Transport Association (IATA) [45] estimates that more than eight thousand cargo aircraft loads are needed to deliver one single vaccine dose to 7.8 billion people worldwide. In Central Europe, however, the majority of vaccines can be transported by land. The environmental costs of transportation [46] from the CO_2_-emitting planes and trucks needed to distribute vaccines from factories to wholesalers, vaccination centers and doctors’ offices to millions of people appear to account for most of the COVID-19 footprint. 

Based on the assumptions in Table 1 and Table 3, the immense influence of transport routes on the carbon footprint becomes evident. Combustion of 1 kg of octane produces 3.088 kg of CO_2_. The CO_2_ equivalent includes greenhouse gases, carbon monoxide, volatile hydrocarbons, nitrogen oxides and particulates. CO_2_ emissions of air travel are based on fuel consumption per person, depending on the distance from takeoff to landing, aircraft type, seating, load factor and cargo carried. One kilogram of kerosene generates around 3.15 kg of CO_2_. Climate-relevant nitrogen oxides and particulate emissions are converted to the climate impact of CO_2_. The radiative forcing index (RFI = 3 to 4) according to the IPCC weighs the theoretically emitted amount of CO_2_ to represent the combustion mixture in the engine. The increased climate impact (contrails, ozone layer) of long-haul flights of 400 km or more at altitudes above 9 km counts as a factor of 3.0 compared to short-haul flights (RFI = 1).

### 3.4. Last Mile Analysis

The last mile is currently considered the most expensive, least efficient and most polluting part of the entire logistics chain [56]. From a sustainability perspective, it is more efficient for trucks to deliver to a few centers equipped with large cold storage facilities than to a large number of small sites that have a short window of time to use the vaccine once it has thawed. Suboptimal logistics and improper refrigeration can also lead to vaccine waste and losses from unused opened vials.

In Germany, the distances between distribution centers, pharmacies and doctors’ offices are relatively short. A sufficient number of regional supply chains allows quick access to several neighboring countries and reduces the overall transport demand. COVID-19 vaccines are shipped as additional load, according to an April 2021 bulletin from the Pharmacy Wholesaler Association.

A simulation was developed to assess the total effort required to distribute about 6.3 million vaccine doses from 41 named hubs to 35,000 general practitioner (GP) practices via 15,000 pharmacies. Missing information about the relationship of physicians to pharmacies and pharmacies’ ordering habits was replaced by a number of assumptions. The distance between hub and pharmacy and between pharmacy and GP practice, respectively, proved to be the most important factor. To make the simulation as relevant as possible, market shares of the wholesalers were considered, too. Ten wholesalers (five nationwide and five regional wholesalers) participate in the distribution, with regional competition in almost all parts of Germany.

**Figure 2:**
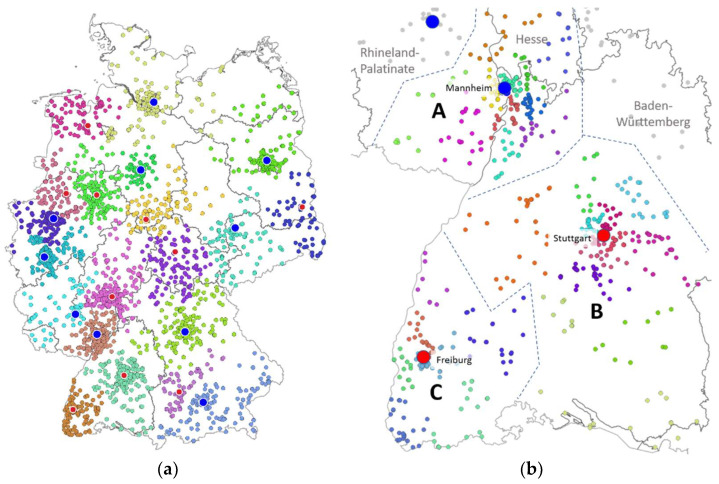
Last-mile analysis for the distribution of mRNA vaccines in Germany in 2021. (**a**) Hub locations and assignments of pharmacies to wholesaler hubs in Germany (simulation result for one wholesaler). Blue: vaccine receipt-points, red: intermediary distribution hubs. (**b**) Pharmacies organized in delivery clusters: simulated example for three hub areas A, B, C of one wholesaler in southwest Germany (Baden-Württemberg, Rhein-Neckar area and Palatinate).

The simulation was based on three tiers: (a) the wholesaler tier with 41 hubs as vaccine receipt-points and 69 hubs serving as intermediary delivery points, (b) the pharmacy tier, organized in ‘delivery clusters’ of 15–30 neighboring pharmacies being served on tours from/to the wholesaler hubs and (c) the GP tier being served on tours from/to the pharmacies. Figure 2 illustrates the simulation results for layers (a) and (b) for one specific wholesaler.

Tours on tier levels (b) and (c) were optimized in this model by generating solutions using the ‘traveling salesperson problem’ (TSP) algorithm [57]. Touring distances between hubs and pharmacies or pharmacies and GP practices were initially calculated as a straight line. Straight-line distances were multiplied by a factor 1.3 (based on experience) to resemble road distances as a replacement for exact routing. For each pharmacy cluster, the traveling salesperson algorithm determines a path that covers all pharmacies almost optimally. A speed of 90 km/h is assumed for the distribution within the wholesalers’ hub network, 60 km/h is assumed for the tour hub – pharmacy cluster – hub and 40 km/h is assumed for the delivery route from the pharmacies to the GP practices. The simulation and the analysis of results was performed in R, version 4.0.5 [58].

Simplified time considerations take into account the separation of pallets by preparing smaller package sizes for pharmacies, loading at the hub (20 min), reloading at intermediary hubs (10 min), touring within the wholesaler network (195 km two-way plus 10 min reloading), tours to pharmacies (224 km, 112–200 min), with stops at pharmacies (10 min each, 80–150 min in total), preparation at the pharmacy (30 min) and delivery to GP practices including stops (6 km, 10 min per stop, 18–65 min in total). On average, a model tour covers 425 km with 284 to 563 min (4.5–9 h) of delivery time. 

The model demonstrated 1.2 million km of driving per month required for the distribution of vaccines to GP practices in Germany. Approximately 370,000 kg CO_2_ equivalents per month would be generated assuming a separate distribution system for vaccines; however, additional tours for vaccine distribution are the exception. The logistics chain for vaccines is typically part of the existing delivery tours. Considering the negligible weight of vaccines as payload, the ecological impact of transportation reduces to 3600 kg CO_2_ equivalents/month—about 9 months of constant driving with one single car. Details are compiled in Table 4.

### 3.5. Carbon Footprint per Dose and Error Estimation

The individual contributions of production, transport and storage to the carbon footprint of one million doses of two mRNA COVID-19 vaccines are summarized comparatively in Table 5. The calculated results of this environmental assessment are an approximation and should not be interpreted as absolute figures. Comparative CO_2_ values for the production, use and disposal of goods are subject to significant uncertainties. It is generally impractical to quantify in detail all the process steps from the raw materials to the end use in order to make a reliable statement about the entire recycling chain. 

The environmental burden of packaging, storage and deep freezing is less than that of air and freight transport. Key drivers of CO_2_ footprint differences are the emissions caused by transporting different weights per vial of vaccines. The impact of airfreight shows the most notable differences in carbon footprint between mRNA vaccines. Land transportation differences are also shown to be impacted by the additional 23 kg of dry ice required per shipment unit of BNT162b2.

Comparing one million doses of two mRNA vaccines in Germany results in a difference of about 1100 kg CO_2_ equivalents (without transportation) and 54,000 kg CO_2_ equivalents (including all modes of transport up to the last mile), respectively. The difference is based on the packaging weight and the number of doses per vial. 

The large contribution of transport determines the statistical uncertainty of the total calculated carbon footprint. For Scenario A, for example, the cumulative individual errors for production, storage and disposal account for only 0.7% of the total error (Equation (1)):

(1) $$\Delta m\left({\mathrm{CO}}_{2}\right)=\sqrt{{\left(109203\right)}^{2}+{\left(760\right)}^{2}}\approx \pm 109206\;\mathrm{kg}\;\left(\mathrm{per}\;10^{6}\;\mathrm{doses}\right)$$

The calculation scheme in Table 5 allows the reader to easily obtain their own results for given transport distances and weights. This allows individual estimations on the same numerical basis for countries other than Germany. 

For comparison of the order of magnitudes: the annual carbon footprint of health care is about 546 Mt CO_2_ equivalents in the USA [59], including 79 Mt CO_2_ equivalents for the prescription of drugs. In other words, giving ten billion doses of mRNA vaccine to the world’s population is equivalent to less than 0.4% of the annual carbon footprint of the total health care system in the United States. 

Regarding medical wastes due to COVID-19 (syringes, masks, shields, disinfectants, etc.), it should be said that vaccination avoids corresponding future wastes because vaccines help to overcome the pandemic faster. Such positive effects were not considered in this study.

## 4. Conclusions

In terms of CO_2_ impact, mRNA vaccines do not impose a significant burden on the environment. Compared to the medical, economic and social implications of COVID-19 vaccinations, the ecological impact of manufacture, storage, freezing and distribution in terms of CO_2_ equivalents is small. Positive effects, including that vaccination avoids future medical waste, were not considered in this study.

The carbon footprint of a single mRNA vaccine dose injected into a patient is about 0.01 to 0.2 kg CO_2_ equivalents, depending on the cooling technology and the logistic routes to the vaccination sites in Germany. 

The emissions for air freight, road transportation and last-mile delivery account for up to 99% of the total carbon footprint, which is nearly as 19 times the emissions generated from ultra-deep freeze technologies, the production of dry ice, glass and medical polymers for packaging. 

In assessing the ecological impact of mRNA vaccines in the most populated European country, Germany, there is a small difference in carbon footprint in favor of mRNA-1273 in the order of magnitude of 1.1 to 54 t CO_2_ equivalents per 1 million doses which corresponds to a journey of 5500 to 270,000 km in a car with an internal combustion engine (0.2 kg/km). 

These findings are necessarily qualitative or semi-quantitative, as the underlying variables and assumptions about CO_2_ equivalences are subject to large individual errors. Nevertheless, this study shows the relationships between production, supply and cold chain. 

## Figures and Tables

**Table 1:** Estimated CO_2_ equivalents for production of electricity and goods.

	kg CO_2_	kWh	Ref.
Primary Energy Production:			
1 kWh of electrical energy	0.502		[29]
1 kWh German electricity mix (2019)	0.401		[30]
Manufacture of 1 kg:			
Dry ice	0.15	0.36	[31]
Gas compression: liquefaction of natural gas	0.14	0.35	[32]
Glass *	0.72	2.73	[33]
Paper	0.61		[34]
Polypropylene for medical syringes	1.5		[35]
PET: Cradle-to-grave without and with recycling	3.71 and 1.54		[36]
Steam	136		
Production of a large refrigerator	322		[37]
Life cycle of a refrigerator (15 years, 5340 L, +5...−5 °C)	6100 … 12,000		[37]

* 6784 Mio. t per year. Energy consumption 18.53 TWh/a, 4.88 Mio. t CO_2_.

**Table 2:** Typical carbon footprint of freezers in CO_2_ equivalents.

Cold Storage Device	Wh L^−1^d^−1^	kWh/d	kg CO_2_/d	
Average refrigerator per liter and day	0.15 … 0.2	–	≈0.1	[41]
Average freezer, 600–700 L (−80 °C)	14 … 18	9.4 … 12.3	7 … 9	[42]
Stationary: 159 L (−20 °C… −80 °C)	42	6.7	2.7	[43]
Portable: 11 L (−20 °C… −80 °C)	255	2.8	1.2	[43]
Inefficient ultra-cold freezer: 50 L (−70 °C)	400	20	8	[44]
Energy required for 1 kg water (20 °C to 0 °C)	23	–	–	*
Energy required for 1 kg ice (0 °C to −70 °C)	40	–	–	*

*$Q=m\;c_{p}\;\Delta T$, $c_{p}=4186\;{J\;\mathrm{kg}}^{-1}K^{-1}$ (water), $c_{p}=2060{\;J\;\mathrm{kg}}^{-1}K^{-1}$ (ice), $c_{p}=703{\;J\;\mathrm{kg}}^{-1}K^{-1}$ (glass).

**Table 3:** CO_2_ equivalents for the transportation of goods including climate impact.

Type of Transportation	kg CO_2_/(1000 km kg)	kg CO_2_/L	
Average car	170 … 320	–	
Transport of payload	0.11 … 0.23	–	[47,48,49]
Tank-to-wheel: per liter of diesel	–	2.5	[49]
Well-to-wheel: incl. losses in power plants, refineries, power lines	–	2.94	[49]
Air freight	0.67	–	[50] *
	kg CO_2_/km per person		
Flight: Geneva–Frankfurt, A320 Economy, 500 km	0.17 … 0.20		[51,52,53]
Average for one-hour flight in Germany	0.0922 … 0.214		[54,55]
New York–Frankfurt, A320 Economy	0.21 … 0.26		[49,51]

* Airbus A320: 2700 L kerosene/h produces 2760 kg CO_2_/L or 7452 kg CO_2_/h.

**Table 4:** Distribution of 1 million vials for a demand of 6,300,000 doses in the course of 1 month.

Last-Mile Analysis	Most Likely Scenario No Extra Tours, Vaccine as Additional Load Only	Worst Case Scenario Extra Tours
Participating doctors	–	35,000
Related pharmacies	–	15,000
Delivery clusters	–	846
Pharmacies per delivery cluster	–	18
Number of deliveries	–	4
Tours within wholesaler network (2 × 75 km)	–	111,451
Tours within pharmacy clusters (172 km)	–	756,662
Last-mile tour (pharmacy–GP practice)	–	366,600
Number of vials per month	1,050,000	–
Weight per vial (g)	36.9	–
Weight of cryo case: 112 g/49 vials (g)	40	–
Total loading weight per month (kg)	42,000	–
Average distance (km)	212	–
Total distance per month (km)	849	1,234,714
Freight units (t · km)	35,663	–
CO_2_ emissions of transport	100 g/1000 km	300 g/1000 km
CO_2_ equivalents of 1 million vials (kg)	3566	370,414
kg CO_2_ equivalents per vial	0.0034	0.353

**Table 5:** Attempted estimation: CO_2_ equivalents for transportation and storage of *N* = 1 million doses of mRNA vaccines.

		Reference Data See Table 1, Table 2 and Table 3	Scenario A Dry Ice	Scenario B No Dry Ice	Error in %
		Mass including packaging	6154 kg	2517 kg	
		Volume per 1 million doses without packaging	375 L	469 L	
1	Transport		kg CO_2_		
	Air freight (USA–Europe)	6200 km · 2.3 kg CO_2_/(1000 km kg) · *m*	87,756	35,892	50
	Truck	1000 km · 0.2 kg CO_2_/(1000 km kg) · *m*	1231	403	20
	Last-mile analysis per dose (see Section 3.4)		566	see A	10
	Traffic to the vaccination site: 1 km per patient (arbitrary)	*N* · 1 km · 0.1 kg CO_2_/km	100,000	see A	100
2	Manufacture				
	mRNA biochemical process: arbitrary	30 µg · 10^−3^ ⋅ 500 kg CO_2_/kg · *N*	15	see A	500
	Glass (1 g/dose)	0.72 kg CO_2_/kg · (*N*/1000)	720	see A	5
	Paper, cardboard, ancillary kit (1 g/dose)	0.61 kg CO_2_/kg· (*N*/1000)	610	see A	5
	Syringes and sterilization [60]	2 kg · (1.5 + 0.77) kg CO_2_/kg · (*N*/1000)	4540	see A	5
3	Storage				
	Freezer farm: 6 months at −20… −80 °C (600 L)	180 d · 8 kg CO_2_/d	1440	see A	10
	Dry ice for shipping: 2 × 5 days per cartoon	2 · 23 kg · *N*/5850 · 0.15 kg CO_2_/kg	1180	–	3
	Freezing during transport: 10 days at −40 °C	10 d · 8 kg CO_2_/d	–	80	10
	Refrigerator in regional centers: 2… 8 °C	10 d · 0.1 kg CO_2_/d	10	see A	20
4	Waste disposal				
	Combustion of plastics [61]	*N* · 0.002 kg · (2.41 − 0.65) kg CO_2_/kg	3520	see A	20
	Glass recycling [35]	*N* · 0.001 kg · 0.4 kg CO_2_/kg (Credit)			
	Paper recycling	*N* · 0.02 kg · 0.064 kg CO_2_/kg (Credit)			
	Total per dose (kg CO_2_ equivalents)	including transport	0.202 ± 0.110	0.148 ± 0.102	54
		excluding transport (absolute error rounded up)	0.012 ± 0.001	0.011 ± 0.001	7

Transportation units: A = BNT162B2: 1 carton (36 kg, 89 L) in thermal container (23 kg or 15 L dry ice + 3 kg packaging) = 5 trays = 975 vials (each undiluted 0.45 mL) = 5850 doses (each diluted 0.375 mL). B **=** mRNA-1273: 1 pallet (290 kg, 54 L) = 8 containers = 115,200 doses. 1 container (6.76 L) = 12 cartons. One carton (0.564 L) = 120 vials (each 5 mL) = 1200 doses.

## Data Availability

Additional data are available from the authors.

## References

[1] Mauskopf J., Standaert B., Connolly M.P., Culyer A.J., Garrison L.P., Hutubessy R., Jit M., Pitman R., Revill P., Severens J.L. (2018). Economic Analysis of Vaccination Programs: An ISPOR Good Practices for Outcomes Research Task Force Report. Value Health.

[2] Wiedmann T., Minx J., Pertsova C.C. (2007). A definition of ‘carbon footprint‘, Chap. 1. Ecological Economics Research Trends.

[3] Zheng J., Suh S. (2019). Strategies to reduce the global carbon footprint of plastics. Nat. Clim. Chang..

[4] Teverson R., Peters T., Freer M., Radcliffe J., Koh L., Benton T., McLeod D., Uren S., Elliot R., Fryer P. (2015). Doing Cold Smarter.

[5] Mouneer T., Elshaer A., Aly M. (2021). Novel Cascade Refrigeration Cycle for Cold Supply Chain of COVID-19 Vaccines at Ultra-Low Temperature −80 °C Using Ethane (R170) Based Hydrocarbon Pair. World J. Eng. Technol..

[6] Santos A.F., Gaspar P.D., de Souza H.J.L. (2021). Refrigeration of COVID-19 Vaccines: Ideal Storage Characteristics, Energy Efficiency and Environmental Impacts of Various Vaccine Options. Energies.

[7] Jiang P., Fan Y.V., Klemes J.J. (2021). Impacts of COVID-19 on energy demand and consumption: Challenges, lessons and emerging opportunities. Appl. Energy.

[8] Liu Z., Ciais P., Deng Z., Lei R., Davis S.J., Feng S., Zheng B., Cui D., Dou X., Zhu B. (2021). Near-real-time monitoring of global CO_2_ emissions reveals the effects of the COVID-19 pandemic. Nat. Commun..

[9] Rume T., Didar-Ul Islam S.M. (2020). Environmental effects of COVID-19 pandemic and potential strategies of sustainability. Heliyon.

[10] Nundy S., Ghosh A., Mesloub A., Albaqawy G.A., Alnaim M.M. (2021). Impact of COVID-19 pandemic on socio-economic, energy-environment and transport sector globally and sustainable development goal (SDG). J. Clean. Prod..

[11] Siddique A., Shahzad A., Lawler J., Mahmoud K.A., Lee D.S., Ali N., Bilal M., Rasool K. (2021). Unprecedented environmental and energy impacts and challenges of COVID-19 pandemic. Environ. Res..

[12] FDA Fact Sheet for Healthcare Providers Administering Vaccine (Vaccination Providers), Emergency Use Authorization (EUA) of the PFIZER-BIONTECH COVID-19 Vaccine to Prevent Corona Virus Disease 2019 (COVID-19), 12/2020. https://www.fda.gov/media/144413/download.

[13] EMA Product Information, Pfizer-Biontech Vaccine; Comirnaty Concentrate for Dispersion for Injection COVID-19 mRNA Vaccine (Nucleoside Modified), 12/2020. https://www.ema.europa.eu/en/documents/product-information/comirnaty-epar-product-information_en.pdf.

[14] NHS Standard Operating Procedure, Moving Pfizer-BioNTech Covid-19 Vaccines from an Ultra-Low Temperature Freezer into a Fridge to Haw. https://www.sps.nhs.uk/wp-content/uploads/2020/12/VH6-Moving-Pfizer-BioNTech-Covid-19-Vaccines-from-an-ultra-low-temperature-freezer-into-a-fridge-to-thaw-Issue-1.4-24.12.20.docx.

[15] FDA Fact Sheet for Healthcare Providers Administering Vaccine (Vaccination Providers), Emergency Use Authorization (EUA) of MODERNA COVID-19 Vaccine to Prevent Corona Virus Disease 2019 (COVID-19), 12/2020. https://www.fda.gov/media/144637/download.

[16] EMA Product Information, COVID-19 Vaccine Moderna Dispersion for Injection. https://www.ema.europa.eu/en/documents/product-information/covid-19-vaccine-moderna-product-information_en.pdf.

[17] DailyMed (2020). Moderna COVID-19 Vaccine—cx-024414 Injection, Suspension. Fact Sheet for Healthcare Providers Administering Vaccine (PDF). Food and Drug Administration (Report). https://dailymed.nlm.nih.gov/dailymed/drugInfo.

[18] BioNTech Provides Update on Vaccine Production Status at Marburg Manufacturing Site, Press Release, Mainz, 26 March 2021. https://investors.biontech.de/de/news-releases/news-release-details/biontech-gibt-update-zu-status-der-impfstoffproduktion-der.

[19] Shin R., Searcy C. (2018). Evaluating the Greenhouse Gas Emissions in the Craft Beer Industry: An Assessment of Challenges and Benefits of Greenhouse Gas Accounting. Sustainability.

[20] Sheldon R.A. (1997). Catalysis and pollution prevention. Chem. Ind..

[21] Sheldon R.A., Arends I., Hanefeld U. (2007). Green Chemistry and Catalysis.

[22] Phadke R., dos Santos Costa A.C., Dapke K., Ghosh S., Ahmad S., Tsagkaris C., Raiya S., Maheswari M.S., Essar M.Y., Ahmad S. (2021). Eco-friendly vaccination: Tackling an unforeseen adverse effect. J. Clim. Chang. Health.

[23] (2011). WHO. Sustainability in Vaccine Packaging.

[24] Lee B.-K., Ellenbecker M.J., Moure-Erase R. (2002). Analyses of the recycling potential of medical plastic. Waste Waster Manag..

[25] Thomas G.P. Recycling of Polypropylene (PP), Azo Cleantech 2019. https://www.azocleantech.com/amp/article.aspx?ArticleID=240.

[26] Saadat S., Rawtani D., Mustansar C. (2020). Hussain environmental perspective of COVID-19. Sci. Total Environ..

[27] Van-Doremalen N., Bushmaker T., Morris D.H., Holbrook M.G., Gamble A., Williamson B.N., Lloyd-Smith J.O. (2020). Aerosol and surface stability of SARSCoV-2 as compared with SARS-CoV-1. N. Engl. J. Med..

[28] Zambrano-Monserrate M.A., Ruanob M.A., Sanchez-Alcalde L. (2020). Indirect effects of COVID-19 on the environment. Sci. Total Environ..

[29] Berechnen Sie Ihre Treibhausgasemissionen Mit Dem CO_2_-Rechner. www.umweltpakt.bayern.de/energie_klima/fachwissen/217/berechnung-co2-emissionen.

[30] Entwicklung der Spezifischen Kohlendioxid-Emissionen des Deutschen Strommix in den Jahren 1990–2019. www.umweltbundesamt.de/sites/default/files/medien/1410/publikationen/2020-04-01_climate-change_13-2020_strommix_2020_fin.pdf.

[31] Efficient Use of Compressed Air for Dry Ice Blasting. www.researchgate.net/publication/282633853_Efficient_use_of_compressed_air_for_dry_ice_blasting.

[32] Vatani A., Mehrpooya M., Palizdar A. (2014). Advanced exergetic analysis of five natural gas liquefaction processes. Energy Convers. Manag..

[33] Energiewende in der Industrie—Abschlussbericht zum Arbeitspaket 2a. www.bmwi.de/Redaktion/DE/Downloads/E/energiewende-in-der-industrie-ap2a-branchensteckbrief-glas.pdf.

[34] Papierindustrie Senkt Energieverbrauch und Emissionen. www.papierundtechnik.de/im-blickpunkt/papierindustrie-senkt-energieverbrauch-und-emissionen/.

[35] Hillman K., Damgaard A., Eriksson O., Jonsson D., Fluck L. Climate Benefits of Material Recycling Inventory of Average Greenhouse Gas Emissions for Denmark, Norway and Sweden. https://norden.diva-portal.org/smash/get/diva2:839864/FULLTEXT03.pdf.

[36] Dormer A., Finn D.F., Ward P., Cullen J. (2013). Carbon footprint analysis in plastics manufacturing. J. Clean. Prod..

[37] Life Cycle Assessment of a Commercial Refrigeration System under Different Use Configurations. summerschool-aidi.it/edition-2015/images/ancona2013/articoli/non_presentati/articolo16_np.pdf.

[38] (2005). Winnacker-Küchler: Chemische Technik.

[39] Bettenhausen C. (2020). Short CO₂ supply may complicate COVID-19 vaccine rollout. Chem. Eng. News.

[40] UPS A Guide to Dry Ice Shipping, April 2019. https://www.ups.com/us/en/services/knowledge-center/article.page?kid=art16a454e6661.

[41] Evans J., Foster A., Huet J.-M., Reinholdt L., Fikiin K., Zilio C., Houška M., Landfeld A., Bond C., Schreurs M. Specific energy consumption values for various refrigerated food cold stores. Proceedings of the 24th IIR International Congress of Refrigeration.

[42] TSX Series Ultra-Low Temperature Freezers. assets.thermofisher.com/TFS-Assets/LED/Reference-Materials/txs-series-ultra-low-freezers-green-fact-sheet.pdf.

[43] Stirling Ultracold’s Innovative ULT Freezers Lead Fight to Preserve and Protect Covid-19 Vaccines. www.stirlingultracold.com/covid-19-2.

[44] Gumpas L.A.M., Simons G. (2013). Factors affecting the performance, energy consumption, and carbon footprint for ultra low temperature freezers: Case study at the National Institutes of Health. World Rev. Sci. Technol. Sust. Dev..

[45] International Air Transport Association (IATA) The Time to Prepare for COVID-19 Vaccine Transport Is Now, Press Release No. 70, 9 September 2020. https://www.iata.org/en/pressroom/pr/2020-09-09-01/.

[46] Lave L.B., Griffin W.M., Kutz M. (2008). The economic and environmental footprints of transportation, Chap. 1. Enviromentally Conscious Transportation.

[47] Transport per LKW CO_2_ Belastung Beim Gütertransport per LKW National und International. www.klimanko.de/co%C2%B2-belastung-berechnen/gutertransport/#.

[48] Emissionsdaten. www.umweltbundesamt.de/themen/verkehr-laerm/emissionsdaten#tabelle.

[49] Berechnung von Treibhausgasemissionen in Spedition und Logistik. www.co2-sachverstaendiger.de/pdf/DSLV-Leitfaden%20Berechnung%20von%20THG-Emissionen%20in%20Spedition%20und%20Logistik.pdf.

[50] Nachhaltigkeit 2019 FACTSHEET. www.lufthansagroup.com/media/downloads/de/verantwortung/LH-Factsheet-Nachhaltigkeit-2019.pdf.

[51] CO_2_-Rechner des Umweltbundesamtes. uba.co2-rechner.de/de_DE/mobility-flight[2.

[52] Atmosfair. www.atmosfair.de/de/kompensieren/flug/.

[53] ICAO Carbon Emissions Calculator. www.icao.int/environmental-protection/CarbonOffset/Pages/default.aspx.

[54] Umwelt Bundesamt. www.umweltbundesamt.de/sites/default/files/medien/366/bilder/dateien/tabelle_vergleich-verkehrsmittel-personenverkehr_2019_uba.pdf.

[55] Airbus A319 A320 A321 Technische Daten/Beschreibung. aerotask.de/airbus-a319-a320-a321-technische-daten-beschreibung/#.

[56] Gevaers R., Van de Voorde E., Vanelslander T. (2014). Cost Modelling and Simulation of Last-mile Characteristics in an Innovative B2C Supply Chain Environment with Implications on Urban Areas and Cities. Procedia Soc. Behav. Sci..

[57] Hahsler M., Hornik K. (2020). Traveling Salesperson Problem (TSP). https://CRAN.R-project.org/package=TSP.

[58] R Core Team (2021). R, a Language and Environment for Statistical Computing.

[59] Chung J.W., Meltzer D.O. (2009). Estimate of the Carbon Footprint of the US Health Care Sector. JAMA.

[60] McGain F., Moore G., Black J. (2017). Steam sterilisation’s energy and water footprint. Aust. Health Rev..

[61] Eriksson O., Finnveden G. (2009). Plastic waste as a fuel—CO_2_-neutral or not. RSC Energy Environ. Sci..

